# Harnessing Epigenetic Mechanisms to Overcome Immune Evasion in Cancer: The Current Strategies and Future Directions

**DOI:** 10.7759/cureus.70631

**Published:** 2024-10-01

**Authors:** Rajabikramaditya Panda, Sumithra Mohan, Chitra Vellapandian

**Affiliations:** 1 Pharmacology, Sri Ramasamy Memorial (SRM) College of Pharmacy, Chengalpattu, IND

**Keywords:** cancer immunotherapy, crispr/cas9, dna methylation, epigenetics, histone modifications

## Abstract

Combining epigenetic alterations with cancer immunotherapy offers a promising approach to improve therapeutic outcomes by targeting the complex biology of tumors. Epigenetic mechanisms such as DNA methylation, histone modifications, and non-coding RNAs (ncRNAs) play a dual role in maintaining immune homeostasis and promoting cancer progression. Changes like hypomethylation in tumor cells can contribute to immune evasion and treatment resistance. Advances in epigenetic tools, particularly clustered regularly interspaced short palindromic repeat (CRISPR) and their associated protein (Cas9)-based epigenetic editing, allow for precise gene expression control, opening new avenues for research and therapy. The improved accuracy of CRISPR/dCas9 systems, when paired with appropriate delivery methods such as viral vectors or nanoparticles, has facilitated innovative combination therapies involving immune checkpoint inhibitors and epigenetic drugs. These combinations enhance the immune system's ability to recognize and destroy cancer cells. Despite these advancements, challenges like off-target effects, delivery issues, and resistance remain. Current research is focused on identifying new therapeutic targets, improving delivery systems, and using real-time feedback to refine treatment. Overall, combining immunotherapy with epigenetic modifications holds significant potential for personalized cancer treatment, paving the way for more effective and individualized therapeutic strategies that target both the immune system and the tumor microenvironment. Further development in this area is expected to revolutionize cancer therapy and improve patient outcomes.

## Introduction and background

Cancer immunotherapy represents a cutting-edge method for treating cancer by harnessing and modifying the body's immune system to selectively target and eliminate cancer cells. Unlike conventional treatments like chemotherapy and radiation that directly attack cancer cells, immunotherapy focuses on boosting or restoring the immune system's innate ability to recognize and destroy tumors. This approach encompasses three primary types, each utilizing a unique strategy to enhance the immune system's response to cancer [1]. Monoclonal antibodies aim to bind to specific targets on cancer cells, enabling the immune system to destroy them. Immune checkpoint inhibitors, such as nivolumab (Opdivo®) and pembrolizumab (Keytruda®), work by blocking proteins that usually suppress immune responses, thereby boosting T cells' capacity to attack cancer cells. Chimeric antigen receptor (CAR)-T cell therapy involves reprogramming a patient's T cells to create chimeric antigen receptors (CARs) that can recognize and eliminate cancer cells [2].

Cancer vaccinations, such as the human papillomavirus (HPV) vaccine, have the potential to prevent some cancers by stimulating the immune system to identify and combat cancer cells. Using genetically engineered viruses, oncolytic virus therapy selectively infects and kills cancer cells while triggering the body's defenses against the tumor [3]. Since epigenetic alterations affect gene expression without changing the underlying DNA sequence, they are important for the initiation and progression of cancer. These alterations include shifts in the histone structure, DNA methylation, and non-coding RNAs. Taken together, these modifications affect how genes are regulated and help cause normal cells to become cancerous [4]. The technique of adding a methyl group to cytosine residues in DNA is known as DNA methylation, and it usually results in the silence of genes. Tumor suppressor genes are typically hypermethylated and muted in cancer, whereas oncogenes may become hypomethylated and overexpressed. Aberrant DNA methylation patterns are frequently seen in cancer. These modifications may interfere with regular cellular functions and encourage the growth of cancer [5]. Histone alterations, including acetylation, methylation, and phosphorylation, impact transcription and gene accessibility by modifying the degree to which DNA is encircled by histone proteins. Alterations in histone modification patterns in cancer can lead to the silencing of tumor suppressor genes or the activation of oncogenes. For instance, abnormal histone methylation can silence genes that normally inhibit tumor growth, while excessive histone acetylation can activate genes involved in cell proliferation and survival [6]. Long non-coding RNAs (lncRNAs) and microRNAs (miRNAs) are two examples of non-coding RNAs that are important in cancer. MiRNAs can control post-transcriptional gene expression, and their dysregulation is frequently linked to the emergence of cancer. Malignant transformation, for instance, may be facilitated by the overexpression of oncogenic miRNAs or the downregulation of tumor suppressor miRNAs. Conversely, chromatin remodeling and interactions with transcription factors are just two of the ways that lncRNAs might affect the expression of genes. Changes in lncRNA expression have been connected to the spread and metastasis of cancer [7].

Both epigenetics and immunotherapy can work together to overcome current barriers and improve therapeutic outcomes, investigating their junction is essential to the advancement of cancer treatment. By changing the expression of immune-related genes, such as through modifications to DNA methylation or histones that suppress the expression of antigens essential for immune recognition or modify immune checkpoint molecules, cancer cells frequently use epigenetic modifications to elude immune surveillance [8]. By focusing on these epigenetic modifications, the immune system's capacity to identify and combat tumor cells can be improved or restored. Additionally, because epigenetic changes can affect immune cell activity and the tumor microenvironment, thereby increasing the tumor's susceptibility to immunotherapy, combining epigenetic therapies with immunotherapy can increase the efficacy of cancer treatments. For instance, DNA methylation inhibitors can reactivate tumor antigens that have been silenced, which enhances the ability of immune checkpoint inhibitors or CAR-T cells to recognize and attack cancer cells [9]. Additionally, epigenetic modifications that impact immune response pathways might cause cancer cells to become resistant to immunotherapy; by identifying and addressing these modifications, immunotherapy's durability and response rates can be increased. Tumor epigenetic profiling offers significant insights into the particular alterations influencing immune response and propelling cancer growth. This allows immunotherapy methods to be tailored to the distinct epigenetic profile of each patient. A novel strategy that could result in more precise and potent treatments that target both the immunological and malignant components of the illness is the combination of epigenetic and immunological therapies [10].

This review explores the role of epigenetic modifications in cancer therapy, focusing on their impact on immune evasion and the potential of combining epigenetic therapies with immunotherapy. It examines current clinical trials and the synergistic mechanisms underlying combination therapies. Additionally, the review addresses the challenges and limitations associated with these approaches, providing a comprehensive overview of how integrating epigenetic and immune-based strategies could enhance cancer treatment outcomes.

## Review

Targeting epigenetic modifications for cancer therapy

Epigenetic modifications are essential mechanisms that control gene expression without changing the DNA sequence itself. These modifications are key players in numerous cellular processes, such as development, differentiation, and the response to environmental cues. In the context of cancer, aberrant epigenetic changes contribute to tumor initiation, progression, and resistance to therapy. Grasping these modifications, including DNA methylation, histone alterations, and non-coding RNAs, is crucial for creating new therapeutic approaches that target and reverse abnormal gene regulation in cancer cells.

DNA methylation is an essential epigenetic alteration that plays a key role in controlling gene expression and ensuring genomic stability. It entails attaching a methyl group to the cytosine base of DNA, specifically at CpG dinucleotides [11]. This process plays a vital role in various cellular functions, such as X-chromosome inactivation, suppression of transposable elements, and gene silencing during normal cellular activities.

Throughout development, stable DNA methylation patterns are set, allowing for heritable changes in gene expression without modifying the DNA sequence. In cancer, however, these patterns are frequently disturbed, which can greatly influence tumor progression. Irregular methylation may result in the silencing of tumor suppressor genes, weakening defenses against cancer, or in the activation of oncogenes, which fosters the growth of cancerous cells. Specifically, hypomethylation in oncogene regions may cause their overexpression, while hypermethylation of tumor suppressor gene promoters results in their silencing, allowing unchecked cell proliferation [12].

Histone modifications, which change the structure of chromatin to control gene expression, include acetylation, methylation, phosphorylation, and ubiquitination. Histones that have undergone acetylation have less DNA binding, which allows for an open chromatin state that encourages transcription. Tumor suppressor genes are frequently silenced in malignancies due to reduced acetylation. Depending on the precise location, methylation can either repress or activate genes; aberrant patterns can either silence tumor suppressors or activate oncogenes, which is how cancer is caused. During cell division and DNA repair, phosphorylation alters chromatin; dysregulation of phosphorylation has been related to cancer. Ubiquitination modifies the expression of genes and chromatin; abnormal patterns in these processes aid in the development of cancer [6].

**Figure 1:**
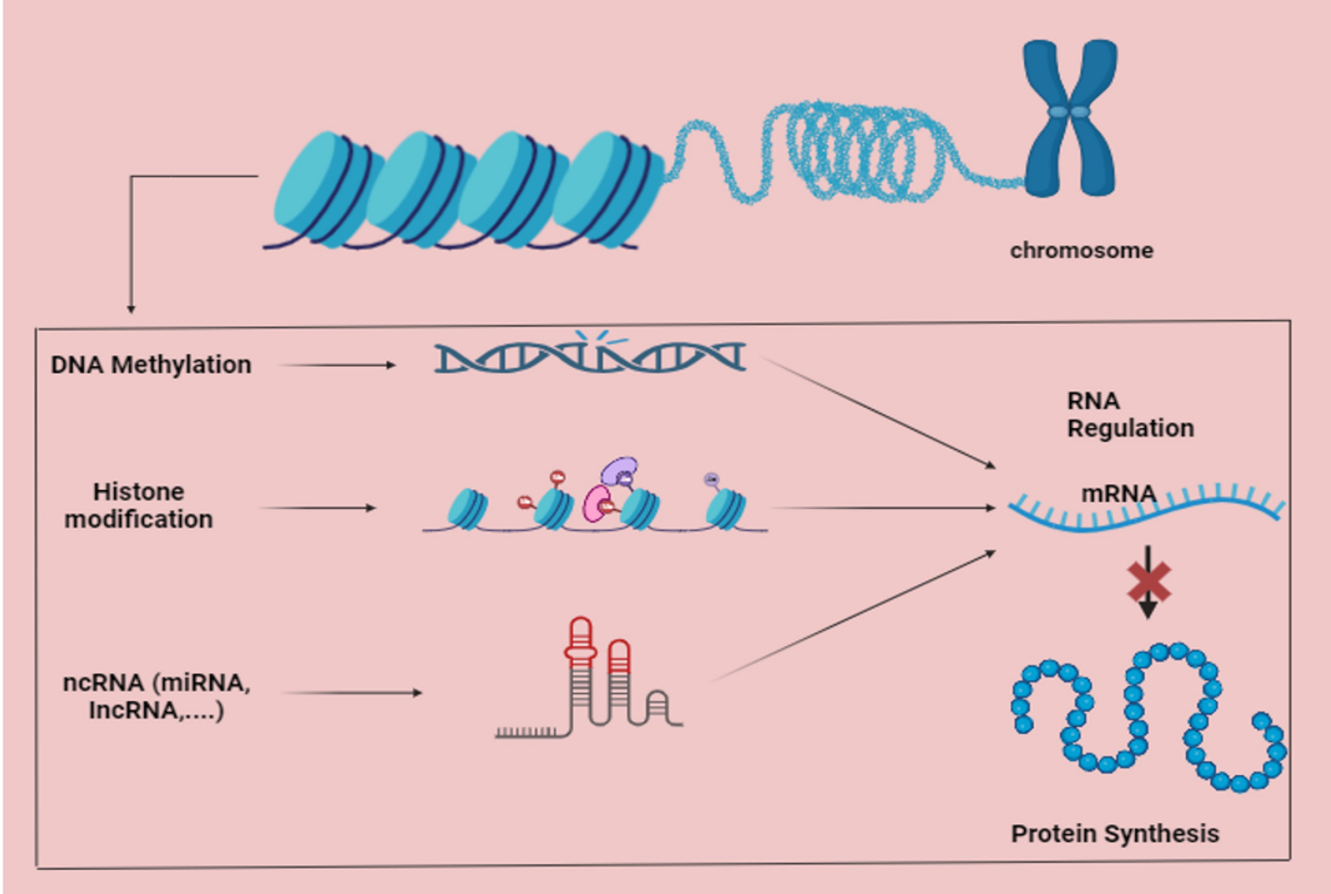
Gene regulation mechanisms The image depicts how histone modification, DNA methylation, ncRNAs, and miRNA function to regulate genes. The RNA regulation that is influenced by these epigenetic mechanisms ultimately impacts the amounts of mRNA. Protein synthesis is impacted by mRNA regulation, which is essential for regulating gene expression and cellular activity. The entire procedure is essential for preserving the integrity of the genome and affecting cellular functions. The figure is the original illustration of the author. It is created using BioRender.com. Image credit: Rajabikramaditya Panda.

lncRNAs and miRNAs are examples of ncRNAs that play a critical role in controlling gene expression and impacting cancer biology, encompassing tumor growth, metastasis, and response to treatment. About 22 nucleotides long, miRNAs control post-transcriptional regulation of gene expression by either preventing translation or directing mRNAs for destruction. They can act as tumor suppressors or oncogenes (oncomiRs) in cancer; two such examples are miR-34 and miR-21. Longer than 200 nucleotides, lncRNAs control the expression of genes using protein interactions and chromatin remodeling, which affects processes like metastasis and proliferation. Notable lncRNAs implicated in the advancement of cancer include HOX Transcript Antisense RNA (HOTAIR*)* and metastasis-associated lung adenocarcinoma transcript 1 (MALAT1*)*. Additionally, ncRNAs have an impact on angiogenesis, metastasis, and immune responses inside the tumor microenvironment (Figure 1). Targeting ncRNAs has therapeutic promise when using strategies like lncRNA inhibitors and miRNA replacement therapy [7].

Epigenetics significantly impacts the tumor microenvironment (TME) by affecting the interactions between tumor cells and adjacent stromal elements like immune cells, fibroblasts, endothelial cells, and the extracellular matrix (ECM). This influence contributes to tumor growth and resistance to therapy. By reprogramming cancer-associated fibroblasts (CAFs) through epigenetic modifications, tumor cells can promote immunosuppression by regulating immune cells and increasing tumor growth, angiogenesis, and metastasis. By modifying the expression of proteins and remodeling enzymes, epigenetic alterations also impact the ECM, promoting the adhesion, migration, and invasion of tumor cells. These changes also affect angiogenesis by controlling the expression of Vascular endothelial growth factor (VEGF) and other proteins that promote tumor vascularization. Epigenetic modifications that affect immune checkpoint molecules like PD-L1 and encourage immunosuppressive TME conditions facilitate tumor immune evasion [13]. Targeting epigenetic changes in the TME provides therapeutic potential to improve treatment efficacy, overcome resistance, and restore the environment, often in conjunction with immunotherapy.

Epigenetic modifications and immune evasion

The process of multiple pathways and epigenetic modifications facilitate tumor immune evasion. Through DNA methylation and histone changes, tumors downregulate major histocompatibility complex (MHC) and tumor-associated antigens (TAAs), decreasing immune cell recognition. Increased DNA methylation is one epigenetic change that can upregulate immune checkpoint molecules such as cytotoxic T-lymphocyte antigen 4 (CTLA-4) and programmed death-ligand 1 (PD-L1), hence increasing immunological suppression and fostering an immunosuppressive environment.

Tumor cells also modify epigenetic processes, leading to increased production of immunosuppressive cytokines such as transforming growth factor beta (TGF-β) and interleukin (IL)-10. This alteration attracts regulatory T cells (Tregs) and myeloid-derived suppressor cells (MDSCs), which impede the formation of an effective anti-tumor immune response. Additionally, these epigenetic changes impact the expression of chemokines and cytokines, altering the inflammatory environment to support tumor survival [14]. Moreover, immune cells' capacity to identify and eliminate tumor cells is diminished by epigenetic modifications brought on by tumors. Lastly, by altering antigen presentation, immune checkpoint expression, or cytokine production, these epigenetic changes may increase resistance to immunotherapies, such as immune checkpoint inhibitors and CAR-T cell treatments. Comprehending these pathways is essential to formulating tactics to surmount resistance and augment the efficacy of immunotherapies [15].

By upregulating immune checkpoint molecules and downregulating the antigen presentation apparatus, tumor cells use epigenetic changes to avoid immune detection. Tumor-associated antigens (TAAs), major histocompatibility complex molecules, and elements of the antigen processing machinery can all be silenced by DNA methylation and histone changes, which lowers antigen presentation and inhibits cytotoxic T lymphocyte activation. To avoid being recognized by the immune system, tumors also employ epigenetic pathways to suppress antigens unique to them. Epigenetic modifications concurrently lead to immune suppression by upregulating the expression of immunological checkpoints such as PD-L1, CTLA-4, and T cell immunoglobulin and mucin domain 3 (TIM-3) through DNA methylation and histone acetylation at promoter sites. An immunosuppressive environment can also be produced by these alterations by activating signaling pathways that upregulate immunological checkpoints, such as the JAK-STAT pathway [16]. Furthermore, epigenetic modifications in immune cells themselves may impact their functionality, rendering them more susceptible to the inhibitory impacts of these checkpoints and impeding the ability of the immune system to effectively combat malignancies.

According to the TME, the tumor epigenetic landscape has a significant impact on immune cell function, which in turn affects tumor growth and therapeutic responses. Epigenetic changes are employed by tumors to alter the behavior of immune cells, hence facilitating immune evasion and growth. Through epigenetic modifications, tumor cells can influence immune cell differentiation. For example, they can boost regulatory T cell's (Tregs) capacity to repress and stimulate the differentiation of myeloid-derived suppressor cells (MDSCs). These changes may also have an impact on the synthesis of chemokines and cytokines, which could result in an imbalance that promotes tumor growth and inhibits immunological responses [17]. Furthermore, immune cells' activation and effector capabilities may be compromised by epigenetic modifications, which would lessen their capacity to identify and eliminate tumor cells. By activating immunosuppressive cells like Tregs and MDSCs and expressing immunological checkpoints like PD-L1, tumors further induce an immunosuppressive TME. Additionally, immune cell activities can be changed by tumor-induced epigenetic reprogramming.

Several groups of epigenetic drugs, such as Enhancer of zeste homolog 2 (EZH2) inhibitors, HDAC inhibitors, and DNA methylation inhibitors, are playing an increasingly important role in the management of cancer. These drugs function by reactivating suppressed genes and preventing tumor growth. Although these agents have shown promise in cancer therapy, they are often associated with hematological and gastrointestinal side effects. Ongoing research is focused on overcoming resistance and optimizing the effectiveness of these therapies in clinical settings (Table 1).

**Table 1:** A summary of epigenetic medication classes, mechanisms, clinical applications, side effects, and therapeutic implications in cancer The summary provided in the table emphasizes the drugs' ability to correct aberrant gene regulation and the ongoing efforts to maximize safety and efficacy. MDS- Myelodysplastic Syndrome, AML- Acute Myeloid Leukemia, CTCL- Cutaneous T-cell Lymphoma, PTCL- Peripheral T-cell Lymphoma, EZH2- Enhancer of Zeste Homolog 2, BET- Bacterial Endotoxins Test, HDAC6- Histone Deacetylase 6.

Epigenetic Drug Class	Drug Examples	Mechanism of Action	Clinical Use	Side Effects	Therapeutic Implications
DNA Methylation Inhibitors	Azacitidine, Decitabine	Incorporate into DNA, bind to DNMTs, prevent methylation, reactivate silenced genes	Hematologic malignancies (e.g., MDS, AML)	Myelosuppression (anemia, neutropenia, thrombocytopenia), gastrointestinal symptoms (nausea, vomiting, diarrhea), injection site reactions, bone marrow toxicity	Effective in reversing abnormal DNA methylation, used in combination therapies, ongoing research to overcome resistance and optimize dosing schedules
Histone Deacetylase (HDAC) Inhibitors	Vorinostat, Romidepsin	Inhibit HDAC enzymes, promote histone acetylation, reactivate silenced genes	Hematologic malignancies (e.g., CTCL, PTCL)	Gastrointestinal symptoms, fatigue, hematological issues (thrombocytopenia, anemia), weight loss, liver enzyme abnormalities, potential cardiac toxicity	Used in combination with other therapies, research is ongoing to refine their use and address resistance
EZH2 Inhibitors	Tazemetostat, EPZ-6438	Inhibit EZH2, reduce H3K27me3 levels, reactivate silenced tumor suppressor genes	Lymphomas, solid tumors	Gastrointestinal symptoms, fatigue, hematological issues (anemia, thrombocytopenia), liver enzyme abnormalities; long-term effects under investigation	Promising in cancers with specific EZH2 mutations, ongoing research to identify new targets and combination therapies
BET Inhibitors	JQ1, I-BET762	Inhibit BET proteins, disrupt transcriptional machinery recruitment	Hematologic malignancies, solid tumors	Gastrointestinal issues (nausea, diarrhea), fatigue, hematological abnormalities	Potential to modulate gene expression in cancers, research ongoing to optimize dosing strategies and combination therapies
DNMT3A Inhibitors	Emerging drugs	Target DNMT3A, reverse aberrant DNA methylation patterns	Early clinical and preclinical studies for cancer	Side effects are still being characterized; may include changes in gene expression and potential impacts on normal cell function	Investigational drugs with potential in cancer therapy, ongoing studies to understand efficacy and safety
HDAC6 Inhibitors	ACY-1215	Inhibit HDAC6, affect protein degradation, cell motility, and tumor growth	Multiple myeloma, other malignancies	Gastrointestinal symptoms, hematological issues, potential impacts on peripheral neuropathy	Research is ongoing to refine their use in combination with other therapies, explore new therapeutic opportunities

Combining epigenetic therapies with immunotherapy

Immune checkpoint inhibitors and epigenetic medications together provide a potentially effective way to treat cancer by boosting anti-tumor immune responses and removing resistance mechanisms. Tumor antigen presentation can be restored by epigenetic medications like DNA methylation and HDAC inhibitors, which increase the immune system's ability to recognize cancers and boost the effectiveness of checkpoint inhibitors. Additionally, by decreasing immunosuppressive cells, they modify the TME and improve the effects of checkpoint inhibitors [15]. When combined with immune checkpoint blockade, these medications can also reactivate tumor suppressor genes that have been silenced and enhance immune cell function, resulting in a more powerful and efficient immune response.

Clinical Evidence

Immune checkpoint inhibitors and epigenetic medications together enhance anti-tumor benefits, including better tumor regression and survival rates in animal models, according to preclinical research. Clinical trials in their early stages for several malignancies, such as solid tumors and lymphoma, are demonstrating encouraging outcomes in terms of higher response rates and longer-lasting responses when certain combinations are employed. On the other hand, difficulties include controlling elevated toxicity and adverse effects, conquering resistance mechanisms, and figuring out the best combination regimens and sequencing tactics. To enhance outcomes, future directions include finding predictive biomarkers, investigating new epigenetic targets, and refining combination methods with other medicines [5].

Case studies and clinical trials exploring these combinations

The AZA-001 Trial

In patients with advanced solid tumors, the AZA-001 trial examined the use of the PD-1 inhibitor pembrolizumab in conjunction with the DNA methylation inhibitor azacitidine. The combination showed encouraging initial activity and was generally well-tolerated; patients showed persistent responses and better progression-free survival when compared to historical controls [18]. These findings imply that azacitidine and immune checkpoint inhibitors together may improve the immune system's capacity to identify malignancies, which could result in improved therapeutic outcomes [19].

The NCT03745716 Trial

In patients with metastatic or incurable solid tumors, the NCT03745716 trial examined the use of pembrolizumab in combination with the DNA methylation inhibitor decitabine. According to preliminary findings, the treatment was well-tolerated with controllable side effects. Additionally, a few patients had partial responses and stable illness, indicating that this combination may enhance checkpoint inhibition's effectiveness. The experiment demonstrates how epigenetic treatments and checkpoint inhibitors can be used together to overcome resistance and improve immunological response.

HDAC Inhibitors and PD-1/PD-L1 Inhibitors Top of Form

The NCT02388715 trials: In patients with recurrent or refractory lymphoma, the combination of pembrolizumab and the HDAC inhibitor romidepsin was investigated in the NCT02388715 study. The mixture showed encouraging results, with some patients seeing full or partial responses and a tolerable safety profile. Notably, there were indications of improved protection against tumors [20]. These findings highlight the possibility of HDAC inhibitors and checkpoint inhibitors collaborating to overcome resistance and enhance therapeutic outcomes in hematologic malignancies [21].

The synergistic mechanisms in combination cancer therapies

Improved Tumor Antigen Presentation

The use of epigenetic drugs, such as DNA methylation inhibitors and HDAC inhibitors, can lead to the re-expression of tumor-associated antigens (TAAs) and major histocompatibility complex (MHC) molecules. DNA methylation inhibitors reverse abnormal DNA methylation, reactivating silenced genes encoding TAAs and MHC molecules, making tumor cells more identifiable to the immune system [12]. HDAC inhibitors enhance histone and non-histone protein acetylation, which also increases the expression of TAAs and MHC molecules. This improved antigen presentation increases the efficaciousness of immune checkpoint inhibitors, like PD-L1/PD-1 inhibitors, by improving their capacity to identify and target tumor cells, leading to a more potent anti-tumor immune response [22].

Modulation of the Tumor Microenvironment

Through the modification of immune cell profiles, chemokine and cytokine expression, and TME composition, epigenetic medicines can bring about changes. Immunosuppressive cells that contribute to an immunosuppressive TME, such as Tregs and myeloid-derived suppressor cells (MDSCs), are fewer in number when HDAC inhibitors are used [23]. DNA methylation inhibitors rewire the TME to promote immune cell infiltration and activity, hence optimizing the milieu for immune checkpoint inhibitors. Immune checkpoint drugs are more effective because of this decrease in TME immunosuppression, which boosts immune cell activity and increases their capacity to target tumor cells [24].

Reactivation of Tumor Suppressor Genes

Because of abnormal DNA methylation or histone changes, many tumor suppressor genes are silenced in cancer cells. By demethylating DNA, DNA methylation inhibitors undo the effects of gene suppression and cause these tumor suppressor genes to become active again. Likewise, histone acetylation is altered by HDAC inhibitors, which similarly causes the reactivation of suppressed tumor suppressor genes. Since tumor suppressor genes are essential for controlling immunological responses and cell development, reactivating these genes increases sensitivity to immune checkpoint inhibition and boosts anti-tumor immunity [25].

Enhancement of Immune Cell Function

Immune cells' epigenetic landscape can be altered by epigenetic medications, improving immune cell activation and function. HDAC inhibitors can modify the activity of dendritic and natural killer (NK) cells while enhancing the cytotoxic function and proliferation of T cells. Meanwhile, BET inhibitors influence the expression of genes related to immune cell activation and function by blocking BET proteins from attaching to acetylated histones. Immune checkpoint inhibitors, which block inhibitory signals on these cells, can further strengthen the immune response against malignancies by improving immune cell activity [26].

Overcoming resistance mechanisms

Immune checkpoint inhibitor resistance in tumors can arise from several pathways, such as modifications to the TME and changes in antigen presentation. By changing the epigenetic state of tumor cells, such as by downregulating immunosuppressive pathways or correcting loss of antigen expression, epigenetic medicines can assist in overcoming some of these resistance mechanisms. For individuals who do not respond well to checkpoint inhibition alone, these medications in combination with immune checkpoint inhibitors can address resistance mechanisms and produce more robust and long-lasting responses [17].

Synergistic effects on cellular pathways

Immune checkpoint inhibitors and epigenetic drugs act on different signaling pathways that regulate tumor growth and immune responses. Epigenetic drugs impact gene expression, cell cycle regulation, and apoptosis, whereas immune checkpoint inhibitors focus on immunological regulatory networks that impede T-cell function. Combining these several pathways to target them all at once can have synergistic effects, increasing the therapeutic efficacy compared to monotherapy due to the increased impact on tumor biology and immune modulation [4].

Challenges and limitations

Although epigenetic medicines show promise in cancer therapy due to their capacity to alter gene expression, safety, and off-target consequences remain a worry. These medications, which include EZH2 inhibitors (like tazemetostat), HDAC inhibitors (like vorinostat and romidepsin), and DNA methylation inhibitors (like azacitidine, decitabine), might unintentionally alter non-target genes or pathways, which can have harmful effects. Modified gene expression and interference with regular cellular processes are possible outcomes of off-target impacts. Hematologic toxicity (such as cytopenias), gastrointestinal toxicity (such as nausea, and vomiting), neurological toxicity (such as peripheral neuropathy), cardiovascular toxicity (such as hypertension), and immune system impacts (such as immune-related adverse effects) are among the dangers that should be taken into consideration. Careful dosing and monitoring, supportive care to control side effects, continuing research to find biomarkers and improve drug design, and combination tactics to boost therapeutic efficacy are all examples of mitigation methods.

Issues with Delivery and Specificity of Epigenetic Therapies

The effectiveness and safety of epigenetic therapies are significantly impacted by the administration and specificity of these treatments. Due to their quick metabolism, poor solubility, or ineffective absorption, many epigenetic medications have limited bioavailability, requiring high dosages and escalating side effects. Obstacles such as the tumor microenvironment and blood-brain barrier make it difficult to achieve targeted delivery, which can result in systemic toxicity and non-specific effects. Effective treatment is further complicated by medication volatility and inadequate cellular absorption. These medications may impact non-target genes and pathways, which could have unforeseen implications and be harmful. As a result, specificity problems occur. Efficacy can also be impacted by resistance mechanisms in tumor cells and variations in epigenetic alterations among patients. Strategies including prodrug methods, targeted drug delivery, nanoparticle-based delivery systems, combination therapies, and personalized medicine are being investigated to improve overall therapeutic results, specificity, and stability of drugs [4].

Resistance Mechanisms to Combined Therapies

The effectiveness of combined therapy, including immune checkpoint inhibitors and epigenetic medicines may be impacted by resistance mechanisms that develop through different paths. Genetic and epigenetic modifications, such as target gene mutations or epigenetic landscape shifts, can modify drug targets or circumvent therapeutic effects, which can result in resistance. The immunosuppressive TME has the potential to reduce the efficacy of both therapies by either upregulating alternative immune checkpoints or increasing the presence of immunosuppressive cells. Increased drug efflux or enhanced drug metabolism are examples of drug efflux and metabolism problems that might lower intracellular drug concentrations and require higher doses [26].

Resistance can also result via cellular plasticity and adaptation, such as the activation of compensatory mechanisms or phenotypic alterations. Furthermore, immune evasion strategies such as changed antigen presentation or increased immunological checkpoint expression can reduce the effectiveness of therapy. Combination therapies to target multiple pathways, sequential therapies to address resistance, treatment personalization based on tumor characteristics, development of novel agents, and monitoring and adaptation of therapies based on resistance patterns are some strategies to overcome these obstacles [2].

Future direction

Novel epigenetic targets in cancer immunotherapy present promising opportunities to enhance current treatments and overcome resistance. Inhibiting enzymes like EZH2 can reactivate tumor suppressor genes, as demonstrated by tazemetostat, while BET protein inhibitors such as JQ1 and CPI-0610 modulate immune checkpoint molecules, reducing tumor-induced immunosuppression. Targeting DNMT3A restores normal methylation patterns and improves antigen presentation, and modulating histone acetyltransferases (HATs), histone deacetylases (HDACs), sirtuins, lysine-specific demethylase 1 (LSD1), polycomb repressive complex 1 (PRC1), and ten-eleven translocation (TET) enzymes can further enhance immune responses and restore tumor suppressor gene expression. Advances in clustered regularly interspaced short palindromic repeat (CRISPR) and their associated protein (Cas9)-based epigenetic editing, combined with small molecule inhibitors like EZH2 or HDAC inhibitors, allow precise gene and epigenetic regulation, offering the potential for cancer and genetic disorder treatments. However, delivery challenges persist, though innovations like viral vectors, nanoparticles, and lipid nanoparticles improve bioavailability and target specificity. Strategies such as biomarker monitoring, adaptive therapy, and optimized drug formulations enhance efficacy and reduce off-target effects while exploring new small molecules, RNA-based therapeutics, and alternative epigenetic modulation techniques to address resistance and offer innovative treatment options.

## Conclusions

Recent developments in epigenetic modification, especially about CRISPR/Cas9-based instruments, have greatly improved cancer immunotherapy by permitting accurate targeting of particular genes and epigenetic markers. Modern delivery methods like viral vectors and nanoparticles, together with breakthroughs like CRISPR/Cas9 for gene activation or repression, have significantly increased the specificity and efficacy of treatments. Immune checkpoint inhibitors and epigenetic medications, such as DNMT and HDAC inhibitors, have been shown to work synergistically to overcome resistance mechanisms and enhance therapeutic outcomes. Furthermore, additional routes for intervention are provided by newer epigenetic targets like as EZH2, BET proteins, and TET enzymes.

Subsequent investigations ought to concentrate on refining these technologies to tackle obstacles including side effects, restrictions on distribution, and resistance mechanisms. To improve treatment efficacy, enhanced specificity and targeting through improved epigenetic targets and delivery systems development will be essential. Furthermore, investigating different methods of epigenetic modification, like RNA-based treatments and small molecule inhibitors, can enhance current strategies. Personalized treatment plans, flexible schedules, and real-time monitoring tools will all improve clinical practice by increasing accuracy and effectiveness. Combining epigenetic modification with immunotherapy has the potential to revolutionize the way cancer is treated by reprogramming the tumor microenvironment, enhancing patient outcomes, providing more robust and effective responses, and opening the door for novel, individualized therapeutic approaches.
